# *VvBAP1* Is Involved in Cold Tolerance in *Vitis vinifera* L.

**DOI:** 10.3389/fpls.2018.00726

**Published:** 2018-06-18

**Authors:** Lixia Hou, Guangke Zhang, Fanggui Zhao, Dan Zhu, Xinxin Fan, Zhen Zhang, Xin Liu

**Affiliations:** Key Laboratory of Plant Biotechnology of Shandong Province, College of Life Science, Qingdao Agricultural University, Qingdao, China

**Keywords:** *Vitis vinifera*, low temperature, *VvBAP1*, soluble sugar, antioxidant enzyme

## Abstract

The majority of commercial grape cultivars originate from the European grape. While these cultivars have excellent organoleptic qualities, they suffer from a relatively poor tolerance to the cold experienced during winter, resulting in significant damage to grapevines. Thus, low temperature is one of the bottlenecks that restrict the further growth of the grape industry. Research on the mechanism of cold tolerance in grapes is therefore very important. BON association protein 1 (BAP1) is a recently discovered phospholipid-binding protein. In *Arabidopsis*, the expression of *AtBAP1* can be regulated via low temperature; however, the function of *BAP1* in the grapevine has not been reported. The *VvBAP1* gene was cloned in our previous studies in grapes, and bioinformatics analysis showed that it harbors the conservative calcium-dependent C2 protein domain. However, little is known about its function and underlying mechanism. In this study, cold treatment was applied to the cold-resistant grape varieties ‘F-242’ and ‘Zuoyouhong’ as well as to the cold-sensitive grape varieties ‘Cabernet Sauvignon’ and ‘Chardonnay.’ The expression level of *VvBAP1* in the cold-resistant varieties was significantly higher than in the cold-sensitive varieties, indicating that *VvBAP1* could be associated with the cold response processes in the grapevine. Using the cold-resistant grape variety ‘F-242’ as material, with the 4°C and CaCl_2_ treatment, the relative expression of *VvBAP1* was determined via qRT-PCR. Both low temperature and low-temperature signal Ca^2+^ induced *VvBAP1* expression. In addition, the *VvBAP1* gene was cloned and transferred into *Arabidopsis* to generate *VvBAP1* overexpressing plants. Biochemical assays and gene expression analyses were conducted on plants subjected to low temperature treatments (4 and -8°C). The obtained results showed that the activities of superoxide dismutase and peroxidase in these transgenic plants were higher than those in wild type (WT) plants, and that cell membrane permeability and malondialdehyde content were both lower compared to WT plants. Furthermore, the content of soluble sugars and the expression levels of sugar-metabolizing related genes, such as *BAM4-7*, *SS4*, and *G6PD5*, were significantly higher than those of WT plants. Furthermore, the expression of low temperature response signal genes, including *CBF1, CBF3*, *COR15a*, *COR6.6*, *COR27*, and *KIN1*, were also enhanced. In summary, these results showed that *VvBAP1* could strengthen the cold resistance in the grapevine through adjusting and controlling the sugar content and activating antioxidant enzyme activity.

## Introduction

*Vitis vinifera* L. is sensitive to low temperature. The major species of grapes cultivated in China is the European grape, which has poor cold resistance (Yu et al., 2017). Consequently, freezing temperatures in winter and cold snaps during spring exert strong negative impacts on the grape production. Therefore, it is of great theoretical and practical importance to explore the mechanism of cold resistance and to improve it in susceptible genotypes.

Apart from the water availability, low temperature is the most important environmental factor limiting the productivity and geographical distribution of plants across the world (Theocharis et al., 2012). In recent research, there is a complex signal transduction system in plants responsing to low temperature. As a result of exposure to low temperatures, many physiological and biochemical cell functions are changed. Often, cell membrane is modified. Then, cytosolic Ca^2+^ is accumulated. And Ca^2+^ could increase levels of ROS and the activation of ROS scavenger systems, changes in the expression of cold-related genes and transcription factors, alterations in protein and sugar synthesis, proline accumulation, and biochemical changes that affect photosynthesis (Thomashow, 1999; Theocharis et al., 2012). These changes maintain normal metabolism and enhance cold tolerance in plant.

In previous studies, several grape genes, regulated by transcription factors, related to low temperature were cloned and their expression profiles were analyzed. For example, the transcription factor genes *C-repeat Binding Factor 1a* (*VrCBF1a*), *VrCBF1b*, and *VvCBF1–VvCBF4* (Xiao et al., 2006, 2008) have been isolated from *V. riparia* and *V. vinifera*. The transcription factor genes *VpWRKY1* and *VpWRKY2* have been cloned from *V. pseudoreticulata* Baihe-35-1, and overexpression of *VpWRKY2* showed improved cold resistance in *Arabidopsis* (Li et al., 2010). *MrWRKY30* has been cloned from *Muscadinia rotundifolia* (Jiang et al., 2015). *MrWRKY30* transgenic *Arabidopsis* plants also showed enhanced tolerance to cold stress accompanied by upregulation of *AtCBF1*, *AtCBF3*, *AtICE1*, and *AtCOR47*. The transcription factor genes *VaICE1* and *VaICE2* have been cloned from *V. amurensis* (Xu et al., 2014b). Overexpression of either *VaICE1* or *VaICE2* in *Arabidopsis* increased the freezing tolerance in non-acclimated plants. Similar results were found for *V. vinifera* (Li et al., 2014). Two putative bHLH transcription factors, named *VvICE1a* and *VvICE1b*, were isolated from table grape cultivar ‘Muscat Hamburg’ (*Vitis vinifera*). The expression of *VvICE1a* and *VvICE1b* were induced by many abiotic stresses, including cold, exogenous abscisic acid (ABA), drought, salinity, and cold-drought conditions. The constitutive expression of *VvICE1a and VvICE1b* improved the tolerance to cold in transgenic *Arabidopsis*. The transcript levels of two stress-responsive genes *AtRD29A* and *AtCOR47* were enhanced under normal growth conditions. *VaERF057* gene, an ET signaling downstream gene, was cloned from *V. amurensis.* The expression of *VaERF057* was strongly induced by low temperature. The overexpression of *VaERF057* also enhanced the cold tolerance of *Arabidopsis*. Under cold treatment, malondialdehyde content was lower and superoxide dismutase, peroxidase, and catalase activities were higher in transgenic lines than in wild-type plants (Sun et al., 2016). *VaPAT1*, a GRAS gene from *V. amurensis* was isolated. The transcription of *VaPAT1* was markedly induced by various abiotic stress treatments such as cold, drought, and high salinity. Overexpression of *VaPAT1* increased the cold, drought, and high salinity tolerance in transgenic *Arabidopsis*. When compared with wild type (WT) seedlings, the *VaPAT1*-overexpressing lines accumulated higher levels of proline and soluble sugar under these stress treatments (Yuan et al., 2016). *VaERD15* was related to cold tolerance in the Chinese wild *V. amurensis* ‘Heilongjiang seedling’ (Yu et al., 2017). Plants that over-expressed *VaERD15* showed higher cold tolerance and accumulation of proline, soluble sugars, proteins, malondialdehyde, and three antioxidant enzymes. A few non-transcription factors were also reported in cold resistance. For example, VaCPK20 (Dubrovina et al., 2015), VaCPK21 (Dubrovina et al., 2016), and VaCPK29 (Dubrovina et al., 2017) are kinases that were upregulated under cold stress. In addition, the aldehyde dehydrogenase genes (*VvALDH11B1* and *VvALDH18B*) (Zhang et al., 2012), *VaPUB* (Jiao et al., 2015, 2017), and *VvIPK2β* (Li et al., 2011) are also non-transcription factors. Although various grape genes that are involved in cold resistance have been identified, understanding of the underlying mechanism is still limited.

BON association protein 1 (BAP1, AT3G61190, GenBank accession number 825291) is a recently discovered phospholipid-binding protein with a C2 protein domain. Only few plant proteins have been reported to include the C2 protein domain, such as AtBON1 (BONZAI1) in *Arabidopsis*. Hua et al. (2001) used AtBON1 as bait to screen and obtain the AtBAP1 protein in a yeast two-hybrid library, and observed that BON1 is necessary for *Arabidopsis* to maintain the plant height at 22°C, and that transferring *35S::BAP1* to *bon1-1* could restore the phenotype in the mutant, demonstrating similar functions for BON1 and BAP2 (Hua et al., 2001; Yang and Hua, 2004). Later, Yang et al. (2007) reported that AtBAP1 restrained programmed cells death in *Arabidopsis* under biotic and abiotic stress (Yang et al., 2007). In addition, the expression of *AtBAP1* can be regulated by multiple stimuli, including low temperature, wounding, and biological stress (Yang et al., 2006; Zhu et al., 2011). The transcription factor AtICE1 can bind to the 35 bp MYC cis-acting element upstream of *AtBAP1*, thus inducing *AtBAP1* expression upon cold stress (Zhu et al., 2011). Nevertheless, BAP1 was not identified in species other than *Arabidopsis* and the biological role of this gene still remains unknown.

In our previous study, the grape gene *VvBAP1* was cloned from the grapevine rootstock ‘F-242’ and bioinformatics analysis showed that VvBAP1 has the conservative calcium-dependent C2 protein domain (Zhang et al., 2014). However, whether *VvBAP1* is involved in the cold tolerance of grapes still remains to be explored. Therefore, we examined the expression of *VvBAP1* in grapevine varieties known to respond differently to cold stress conditions. Then, we overexpressed *VvBAP1* in *Arabidopsis* plants and analyzed the related phenotypes by measuring various physiological indexes associated to cold resistance, to unravel the VvBAP1-mediated mechanisms inducing cold resistance.

## Materials and Methods

### Plant Materials and Growth Conditions

#### Grape Tissue Culture Seedling

‘F-242’ shoots with buds were used as tested explants. They were treated with 75% ethanol for 30 s, 0.1% HgCl_2_ for 8 min, and were rinsed with sterile water 3–5 times. They were inoculated on 1/2 MS + 0.1 mg⋅L^-1^ IAA root media under light intensity of 200 μmol⋅m^-2^⋅s^-1^ (25 ± 1°C, 12 h/12 h light cycle). Under aseptic conditions, rooted test-tube plantlets were inoculated on 1/2 MS + 0.1 mg⋅L^-1^ IAA culture media for root induction. The plants were used in experiments after 30–50 days.

#### *Arabidopsis* Cultivation

*Arabidopsis* seeds were sterilized for 15 min with 10% NaClO, rinsed 5 times with sterile water, and were then sown on sterile MS solid medium for 2 days treatment at 4°C to break dormancy. Seeds were then transferred into illumination incubators (22°C, 16 h/8 h light cycle) for 1 week of vertical growth, then transferred to a culture medium (mixed nutrient soil and vermiculite 1:1) for cultivation, with light intensity of 120 μmol⋅m^-2^⋅s^-1^ and relative humidity of 60% (22°C, 16 h/8 h light cycle).

Generation of *VvBAP1* overexpressing *Arabidopsis* plants *AtBAP1* (AT3G61190) sequence in *Arabidopsis* was carried WU-BLAST alignment to search for grape homologous sequences. Specific primers were designed as follows: pMD-*VvBAP1*-F, 5′-GCTCTAGAAGTATCTACTACATAGTTTGCGTACT-3′, *Xb a*I site underlined, pMD-*VvBAP1*-R, 5′-GGGGTACCTTTT ATTATTTTCTCCGAATTGAC-3′, *Kpn*I site underlined. Total RNA was extracted and cDNA was reverse transcribed via PCR. Amplification was performed at 94°C for 5 min; 30 cycles of 94°C for 30 s, 60°C for 30 s, and 72°C for 1 min; followed by 5 min at 72°C, and storage at 4°C. The PCR amplified fragments were cloned into the pMD18-T vector using the restriction enzyme sites *Xba*I and *Kpn*I. The pMD18-T-*VvBAP1* was constructed Successfully. The *VvBAP1* sequence was submitted to NCBI, and GenBank accession number is JQ658428.1 for *VvBAP1* gene, and GenBank accession number is AFD97530.1 for VvBAP1 protein.

*VvBAP1* cDNA was obtained via reverse transcription as template, sequence was amplified by PCR using the following primers: pSuper1300-*VvBAP1*-F, 5′-GCTCTAGAATGGAGGCC GCTTCAGGG-3′, *Xba*I site underlined, pSuper1300-*VvBAP1*-R, 5′-GGGGTACCTCAAACTTGAACCGTCCGATACGAG-3′, *Kpn*I site underlined. Amplification was performed at 94°C for 5 min; 30 cycles of 94°C for 30 s, 60°C for 30 s, and 72°C for 1 min; followed by 5 min at 72°C, and storage at 4°C. The PCR product was cloned into the *Xba*I and *Kpn* I sites and ligated downstream of the CaMV35S promoter of pSuper1300 vector to create the pSuper1300-*VvBAP1* construct, which was transformed in *Agrobacterium tumefaciens* GV3101 (Hao et al., 2017). *Arabidopsis* transformation was performed using the floral dip method (Clough and Bent, 1998). Seeds were collected from infiltrated plants and selected on 1/2 MS medium supplemented with 50 mg⋅L^-1^ hygromycin B; subsequently, the survivors were confirmed via PCR and qRT-PCR analysis. The homozygous T3 progeny of transgenic seedlings were used for functional studies.

The sequence of genomic DNA was used as template and the *VvBAP1* Promoter was amplified by PCR using the following primers: *VvBAP1* Promoter-F, 5′-AACTGCAGAGAA ACATCGTATCATAAAACAAC-3, *Pst* I site underlined, *VvBAP1* Promoter-R, 5′-CGGAATTCATTAATAAGTACGCAAACTA TGTAG-3′, *EcoR* I site underlined. Amplification was performed at 94°C for 5 min; 30 cycles of 94°C for 30 s, 55°C for 30 s, and 72°C for 1 min; followed by 5 min at 72°C, and stored at 4°C. The promoter of *VvBAP1* was cloned into the *Pst* I and *EcoR* I sites of the pCAMBIA1391 vector to create the pCAMBIA1391-*VvBAP1 Promoter*-GUS construct. The same method was used to obtain the homozygous T3 progeny of transgenic seedlings.

### Plant Material Treatment

‘F-242’ tissue culture plantlets of grapes at the 4–5 weeks phase were treated with low-temperature (4°C) and CaCl_2_ (10 mmol⋅L^-1^) for 0, 3, 6, 9, 12, 18, and 24 h. The leaves of the treated plants were collected and stored in liquid nitrogen.

*VvBAP1 Promoter::GUS* transgenic *Arabidopsis* at the 10 days phase, flowers and fruits of mature plants, and transgenic *Arabidopsis* leaves with *VvBAP1 Promoter::GUS* at the 2 weeks phase were used for low-temperature treatment at 4°C for 0, 3, 6, 9, 12, 18, and 24 h, and for CaCl_2_ (10 mmol⋅L^-1^) treatment. The tissue specificity of *VvBAP1* expression was tested via GUS staining. The histochemical localization of GUS staining in transformed plant organs was assayed according to the methods described by Jefferson (1987).

Wild type and *VvBAP1* transgenic *Arabidopsis* plants at the 10 days phase were used for the cold acclimation treatment: 48 h at 4°C and 16 h at 1°C; then, temperature was decreased by 1°C per hour until -8°C was reached, which was maintained for 2 h. Then, the temperature was increased at a rate of 1°C/h until reaching 4°C, which remained stable overnight. Finally, plants were recovered to cultivation at 22°C for 2–3 days for phenotype observation and data collection of the survival rate.

Wild Type and *VvBAP1* transgenic *Arabidopsis* at the 4 weeks phase were subjected to 0, 12, and 24 h of low temperature treatment at 4°C, followed by 2 and 4 h of freezing treatment at -8°C to test the change in cell membrane permeability, malondialdehyde (MDA) content, superoxide dismutase (SOD) and peroxidase (POD) activities, soluble sugar content, *Cu/ZnSOD* and *PRX34* expression levels, and expression levels of sucrose-metabolizing enzyme genes and genes related to low temperature.

For all of the above-mentioned experiments, we conducted three independent experiments (three biological replications and three technological replications in every independent experiment) and one representative result was displayed.

### RNA Extraction and Quantitative Real-Time PCR

Total RNA was extracted from the leaves of the treated plants using the CTAB methods as presented by Iandolino et al. (2004). Concentration, yield, and quality control indices based on absorbances at 230, 260, and 280 nm (A_260/230_ and A_260/280_ ratios) were performed with 10 μL of resuspended total RNA. Five microliters of the total RNA solution was loaded onto a 0.9% agarose gel, electrophoresed to separate RNA, stained with ethidium bromide (EtBr), and visualized under UV light to investigate the size distribution of the total RNA and the integrity of ribosomal bands. DNAse treatment was applied on total RNA samples prior to retrotranscription.

The first chain of cDNA was synthesized via the M-MLV RT Kit (Promega, United States). Real-time PCR was performed using the MyiQ Real-Time PCR Detection System (Bio-Rad, United States) in the presence of SYBR green I (BioWhittaker Molecular Applications) in the amplification mixture according to the manufacturer’s protocols. The procedures for qRT-PCR were as follows: 60 s at 95°C, 10 s at 95°C, 20 s at 58°C, and 15 s at 72°C in 40 cycles. A melting curve was added at the end of each RT-qPCR reaction (72 to 99°C for 45 s, and 5 s for each 1°C increase) to assess the presence of any non-specific products. Three technical replicates were run per sample. The endogenous *Actin* gene was used to normalize the expression levels of target transcripts. The relative transcript levels were calculated using the 2^-ΔΔCT^ method (Livak and Schmittgen, 2001). Oligonucleotides used in real-time PCR experiments are listed in Supplementary Table 1.

### Activity Measurement of SOD and POD

Activity of SOD and POD was determined using the same methods as in our previous study (Fu et al., 2013). In brief, *Arabidopsis* seedlings were homogenized and centrifuged and the ability of the supernatant to inhibit photochemical reduction of NBT chloride by 50% was considered as one unit of SOD activity. We measured the POD according to the following method (Jiang et al., 2016): a 0.01 increase in absorbance per min at 470 nm resulting from oxidation of guaiacol to tetraguaiacol was considered as one unit of POD activity.

To obtain the activity of each enzyme, the data points are the average of three replicates. Three experiments were performed, and the results remained consistent. The result from one set of experiments is presented here.

### MDA Content and Electrolyte Leakage Measurements

Lipid peroxidation was also estimated by methods used in our previous work (Fu et al., 2013), which measured the concentration of thiobarbituric acid reactive substances. A homogenate of 0.1 g leaves with 1 ml 10% (w/v) trichloroacetic acid (TCA) was centrifuged for 10 min at 4000 rpm. Five hundred microliters of this supernatant was added to 500 μl 10% (w/v) TCA, which contained 0.6% (w/v) thiobarbituric acid (TBA). After 15 min incubation in boiling water and cooling to room temperature, the mixture was again centrifuged for 10 min at 4000 rpm. Absorbance was measured at 450, 532, and 600 nm. The extinction coefficient of 155 nM^-1^ cm^-1^ was used to confirm MDA content, which was expressed as nmol⋅g^-1^ FW.

Treated leaves were rinsed with deionized (DI) water, dried, and disks were obtained with a punching bear before incubation at 25°C DI water for 1 h. A conductivity meter (YSI model 55) was used to measure the electrical conductivity (*EC1*) of the leakage solution. After heating the solution in a 100°C water bath for 10 min and subsequently cooling the solution to 25°C, both total ionic strength and the electrical conductivity (*EC2*) were measured, following the methods reported in Welti et al. (2002). Membrane relative permeability was calculated as follows: EC1/EC2 × 100%.

At least three experiments were performed, and the results remained consistent. The result from one set of experiments is presented here.

### Soluble Sugar Content

Soluble sugar content was determined using a previously published method (Zhu et al., 2018). One gram of fresh leaf tissue was reduced to small pieces and then transferred to a test tube with 15 ml of distilled water. Samples were then incubated in a water bath at 70–80°C for 30 min; after cooling, saturated neutral lead acetate was added drop by drop to remove protein in the mixed liquid until the formation of white precipitate ceased; the mixture and residual was placed in a 100 ml volumetric flask and water was added up to scale. The mixture was filtered with a funnel into a conical flask with a small amount of sodium oxalate powder, to remove excessive lead acetate in the filtrate by forming lead oxalate precipitate. The transparent liquor was filtered to obtain soluble sugar extract for the absorbance test under 620 nm wavelength. At least three experiments were performed, and the results remained consistent. The result from one set of experiments is presented here.

### Measurement of BAM Activity, SS Activity, and G6PDH Activity

β-amylase (BAM) activity measurement was measured as previously described (Hagenimana et al., 1992). The dinitrosalicylic acid (DNS) method was applied to test for BAM activity. The enzyme activity unit was calculated based on hydrolyzed maltose formed in the germinating seed.

Sucrose synthase (SS) activity was detected according to established methods (Miron and Schaffer, 1991). SPS was determined in reaction mixtures (70 μL) containing 50 mmol⋅L^-1^ Hepes-NaOH (pH 7.5), 15 mmol⋅L^-1^ MgCl_2_, 25 mmol⋅L^-1^ Fru, 25 mmol⋅L^-1^ UDPGlc, and 40 μL of extract. Mixtures were incubated for 30 min at 37°C, and incubation was terminated with the addition of 70 μL of 30% KOH. Enzyme blanks were terminated with KOH at 0 min.

Glucose-6-phosphate dehydrogenase (G6PDH) activity was detected according to the Wakao and Benning (2005) method with minor changes. Five grams of fresh cells were precooled and ground into homogenate in 10 ml extraction buffer (in an ice bath); the homogenate was screened by four-layer gauze and centrifuged for 10 min at 3000 rpm. The obtained supernatant formed the enzyme crude extract that was used for testing the enzyme activity. Enzyme crude extract and reaction buffer [55 mM Hepes-Tris (pH 7.8), 3.3 mM MgCl_2_, 0.5 mM D-glucose-6-phosphate disodium salt, 0.5 mM NADPNa_2_] were mixed in a water bath at 30°C. Two milliliters of reaction buffer was added to 0.1 ml of enzyme buffer and 0.1 ml of Tris buffer grade for colorimetric comparison at OD_340_ with an interval of 30 s and scanning for 5 min; the change in absorbance values was recorded to obtain the activity of G6PDH (extinction coefficient as 6.22 mM^-1^ cm^-1^).

For each of the enzyme activities, the data points are the average of three replicates. Three experiments were performed, and the results remained consistent. The result from one set of experiments is presented here.

### Statistical Analysis

One-way ANOVA analysis was conducted on test results based on Data Processing System (DPS) software. Tukey *post hoc* tests were applied (Tang and Zhang, 2012). A difference was considered significant at the 0.05 level (*P* < 0.05). Data are the means ± SE of three independent experiments (three biological replicates and three technical replications for each independent experiment).

## Results

### Expression Pattern of *VvBAP1*

**FIGURE 1:**
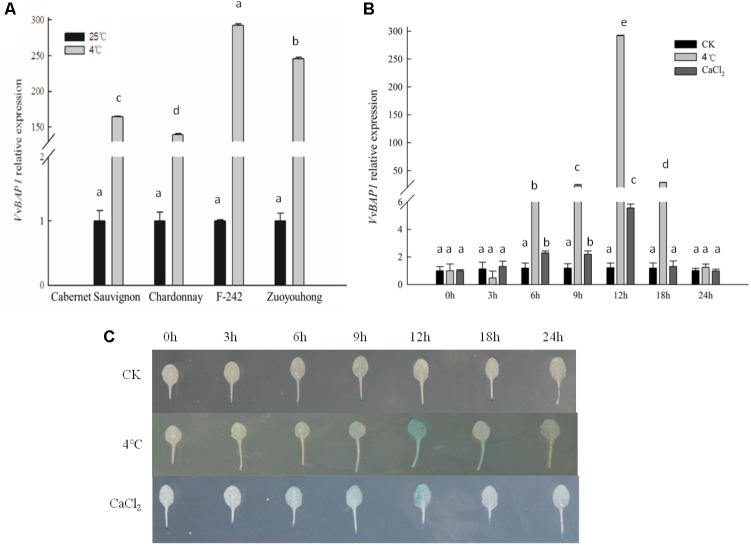
Expression model of *VvBAP1.* **(A)** Difference in *VvBAP1* expression between the cold-sensitive varieties ‘Cabernet Sauvignon’ and ‘Chardonnay’ and the cold-resistant varieties ‘F-242’ and ‘Zuoyouhong’ with 4°C treatment after 12 h. Three independent experimental replications were conducted. Values are the means ± SE of three independent experiments (*P* < 0.05). Lower-case letters above bars denote significant differences attested by Tukey’s HSD test. **(B)** Expression of *VvBAP1* in the cold-resistant varieties ‘F-242’ with the 4°C and CaCl_2_ treatment. Three independent experimental replications were conducted. Values are the means ± SE of three independent experiments (*P* < 0.05). Lower-case letters above bars denote significant differences attested by Tukey’s HSD test. **(C)** GUS enzyme activity in *VvBAP1 promoter::GUS* transgenic *Arabidopsis* with 4°C and CaCl_2_ treatment. GUS stains were repeated three times and at least 30 plants for each individual line were used in each repeated experiment and one representative picture was shown.

To explore the possible correlation between *VvBAP1* and the response to low temperature stress in the grapevine, tissue culture plantlets of the cold-sensitive varieties ‘Cabernet Sauvignon’ and ‘Chardonnay’ and the cold-resistant varieties ‘F-242’ and ‘Zuoyouhong’ at the 4 week phase were used to investigate the expression profiles of *VvBAP1* in the leaves. After 12 h of 4°C treatment, the relative expression of *VvBAP1* in the cold-resistant varieties ‘F-242’ and ‘Zuoyouhong’ was significantly higher than that of the cold-sensitive varieties ‘Cabernet Sauvignon’ and ‘Chardonnay.’ ‘F-242’ showed the highest expression (**Figure 1A**). For this reason, tissue culture plantlets of the cold-resistant variety ‘F-242’ were utilized as materials in a follow-up experiment with 4°C and CaCl_2_ treatment and the relative expression of *VvBAP1* was determined via qRT-PCR. Both low temperature and low-temperature signal Ca^2+^ could induce *VvBAP1* expression, showing the highest expression level at 12 h (**Figure 1B**). The *VvBAP1* promoter fused with GUS transformed into *Arabidopsis* to obtain transgenic plants with GUS staining after 4°C and CaCl_2_ treatment in order to test *VvBAP1* expression profiles. GUS activity reached the maximum level within 12 h, which is consistent with the test results of qRT-PCR (**Figure 1C**). These data suggest that *VvBAP1* is involved in the cold resistance process in grapes.

### *VvBAP1* Enhanced Cold Tolerance in Transgenic *Arabidopsis*

**FIGURE 2:**
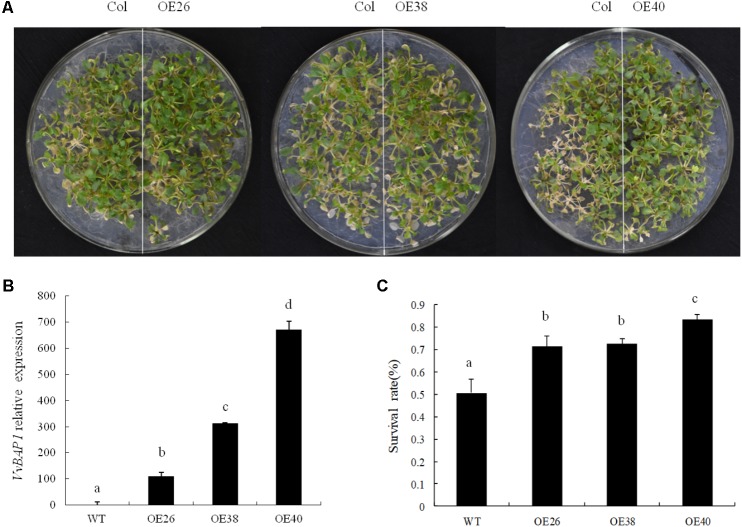
Effects of low temperature on the growth of *VvBAP1* ectopic overexpressing plants. **(A)** Phenotype of transgenic overexpressing lines after the -8°C treatment. These experiments were repeated three times and at least 30 plants for each individual line were used in each repeated experiment and one representative picture was shown. **(B)** Relative expression of *VvBAP1* in different transgenic lines. Three independent experimental replications were conducted. Values are the means ± SE of three independent experiments (*P* < 0.05). Lower-case letters above bars denote significant differences attested by Tukey’s HSD test. **(C)** Survival rates of overexpressing plants after -8°C treatment. Three independent experimental replications were conducted. Values are the means ± SE of three independent experiments (*P* < 0.05). Lower-case letters above bars denote significant differences attested by Tukey’s HSD test.

To further explore the mechanism with which *VvBAP1* improves cold resistance in plants, this gene was transferred to WT *Arabidopsis* and *VvBAP1* over-expressing *Arabidopsis* were identified via PCR (Supplementary Figure 1). And then the relative expression of *VvBAP1* in the obtained ectopic overexpressing lines were tested. Three lines with different expression levels (OE26, OE38, and OE40) were selected for a follow-up experiment (**Figure 2B**). WT and transgenic *Arabidopsis* at the 10 days phase were subjected to 48 h of cold acclimation at 4°C and 16 h of 1°C, followed by a temperature reduction to -8°C at a rate of 1°C/h in 2 h, and a subsequent temperature increase to 4°C at a rate of 1°C/h, followed by a night in dark conditions. They were recovered to cultivation at 22°C for 2–3 days for phenotype observation. The growth of three transgenic lines was higher than to that of the WT *Arabidopsis*, with the OE40 line showing the highest cold resistance (**Figure 2A**). The survival rates of ectopic overexpressing plants were 71, 72, and 83%, compared to 51% in the WT variety (**Figure 2C**), showing that *VvBAP1* could improve the cold tolerance of plants.

### Analysis of Physiological Indexes Related to Cold Resistance of *VvBAP1* Transgenic *Arabidopsis* Plants

**FIGURE 3:**
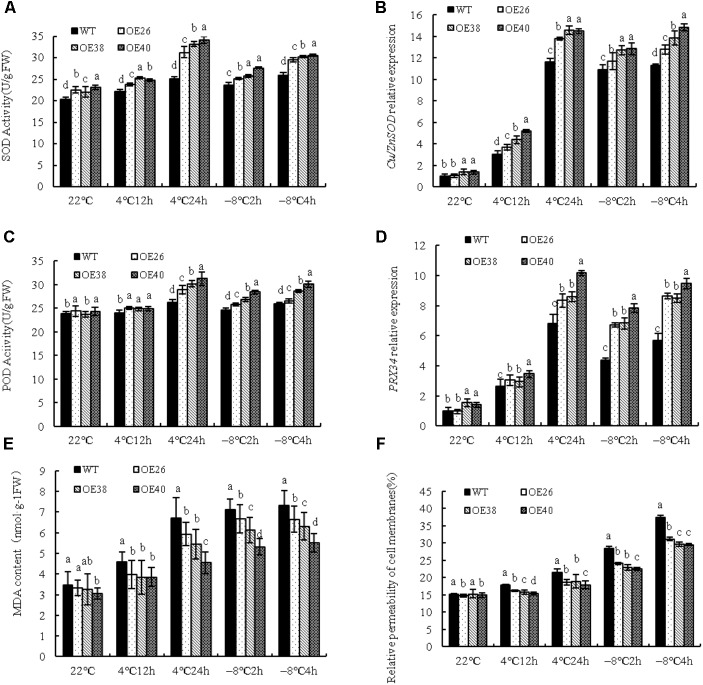
Analysis of physiological indexes of *VvBAP1* transgenic *Arabidopsis* plants in low temperature. SOD activity **(A)** and *Cu/ZnSOD* expression **(B)**, POD activity **(C)**, *PRX34* expression **(D)**, MDA content **(E)**, and cell membrane permeability **(F)** in the leaves of overexpressing lines with the 4°C and -8°C treatment. Three independent experimental replications were conducted. Values are the means ± SE of three independent experiments (*P* < 0.05). Lower-case letters above bars denote significant differences attested by Tukey’s HSD test.

Under cold stress, plants activate their antioxidant protective system to prevent the over-accumulation of reactive oxygen species (ROS). The activities of SOD and POD were tested in transgenic plants to investigate this point, and they both significantly increased after low-temperature treatment at 4 and -8°C (**Figures 3A,C**). The increased levels of *Cu/ZnSOD* and *PRX34* show that relative expression was increased (*P <* 0.05) (**Figures 3B,D**). The enzymatic activity and genetic expression of over-expressing plants were significantly higher than those of WT varieties.

Malondialdehyde, the product of membrane lipid peroxidation, also reflects the degree of plant damage. *VvBAP1* overexpressing plants at the 4 weeks phase were used with 4 and -8°C treatments to detect the change of MDA content. The MDA content was significantly increased after cold treatment at 4 and -8°C; however, the change of MDA content in over-expressing plants was significantly lower than that of the WT (**Figure 3E**). Low temperature can induce an enhancement of cell membrane permeability. The results showed that the cell membrane permeability of over-expressing plants was significantly lower than that of WT plants (**Figure 3F**). This provides further evidence that *VvBAP1* plays an important role in enhancing cold resistance in plants.

### *VvBAP1* Is Involved in Cold Tolerance by Promoting the Accumulation of Soluble Sugars

**FIGURE 4:**
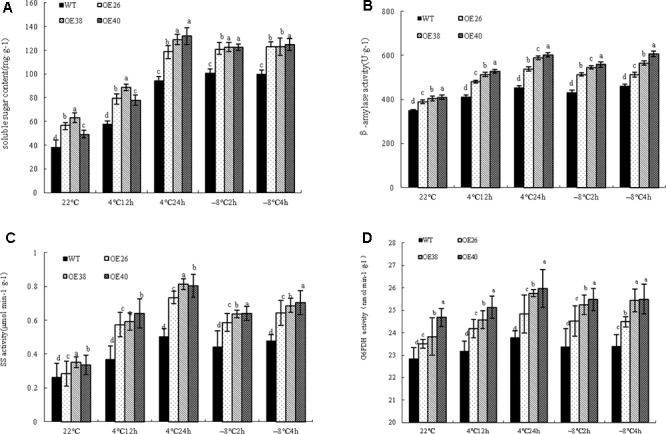
Effects of low temperature on expression of low temperature related genes of *VvBAP1* ectopic overexpressing plant leaves. The soluble sugar content **(A)**, the enzyme activity of BAM **(B)**, SS **(C)**, and G6PDH **(D)** in the leaves of overexpressing lines with the 4 and -8°C treatment. Three independent experimental replications were conducted. Values are the means ± SE of three independent experiments (*P* < 0.05). Lower-case letters above bars denote significant differences attested by Tukey’s HSD test.

Soluble sugars as osmotic adjustment substances play an important role in the plant response to cold stress (Markovskaya et al., 2010). The soluble sugar content in cells is significantly increased under cold stress, which reduces the freezing point of cells. An interesting phenomenon was found during the growth process of *VvBAP1* overexpressing *Arabidopsis* plants: more aphids were found on the stems and leaves of transgenic plants compared to WT (Supplementary Figure 2), which is probably due to the high soluble sugar content in transgenic lines. To prove the effect of *VvBAP1* on improving cold resistance through an increase in soluble sugar content, *VvBAP1* over-expressing *Arabidopsis* at the 4 weeks phase were subjected to a cold treatment at 4 and -8°C. The soluble sugar content in transgenic plants was significantly higher than that of WT plants under cold stress (**Figure 4A**). The activities of key enzymes related to the metabolism of soluble sugars in plants, including BAM (**Figure 4B**), SS (**Figure 4C**), and G6PDH (**Figure 4D**), were significantly higher in transgenic plants than in WT plants.

**FIGURE 5:**
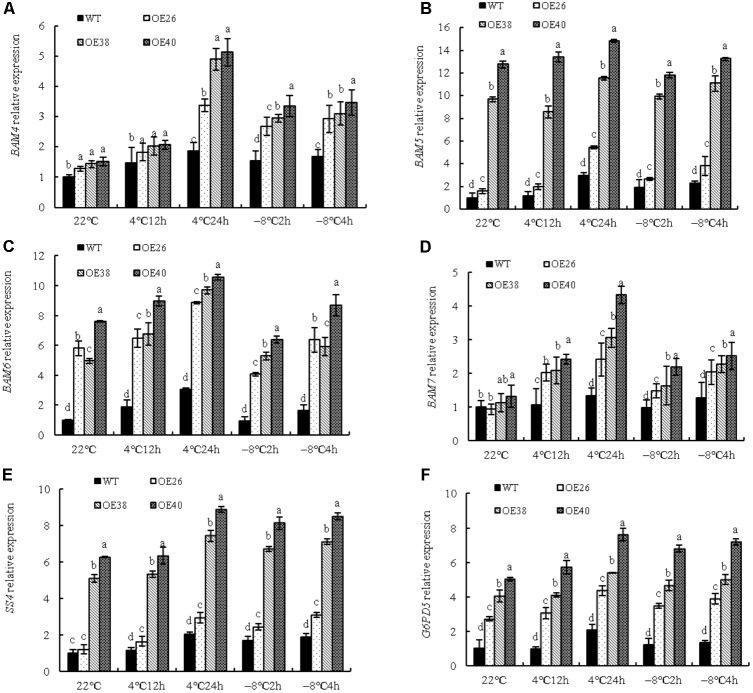
Effects of low temperature on relative expression of genes encoding sugar metabolism enzymes of *VvBAP1* ectopic overexpressing plant leaves. Relative expression of genes encoding sugar metabolism enzymes *BAM4* **(A)**, *BAM5* **(B)**, *BAM6* **(C)**, *BAM7* **(D)**, *SS4* **(E)**, and *G6PD5* **(F)** in the leaves of overexpressing lines with the 4 and -8°C treatment. Three independent experimental replications were conducted. Values are the means ± SE of three independent experiments (*P* < 0.05). Lower-case letters above bars denote significant differences attested by Tukey’s HSD test.

To further corroborate that *VvBAP1* could adjust the soluble sugar content under low temperature, the expression of genes related to sugar metabolism enzymes were detected. The expression of *BAM4*, *BAM5*, *BAM6*, and *BAM7*, of the nine tested members in the *BAM* gene family (**Figures 5A–D**), were significantly increased. Both *BAM4* and *BAM7* were highly activated at 4°C but specifically after 24 h of treatment, whereas the *BAM5* and *BAM6* genes were overall induced in the tested conditions. Furthermore, *BAM5* and *BAM6* are more abundant among analyzed samples. While, the expression of *BAM1*, *BAM2*, *BAM3*, *BAM8*, and *BAM9*, were not significantly changed (Supplementary Figure 3). In addition, the expression of *SS4* (**Figure 5E**) and *G6PD5* (**Figure 5F**) was increased, along with the relative expression of *VvBAP1*. The *SS4* and *G6PD5* genes were all induced in the tested conditions. It was presumed that *BAM5*, *BAM6*, *SS4*, and *G6PD5* might maintain the high sugar content during normal growth of transgenic lines. Furthermore, these six genes all could help to support the increase in sugar content observed under cold stress.

### *VvBAP1* Is Involved in Cold Tolerance by Enhancing Cold-Related Gene Expression

**FIGURE 6:**
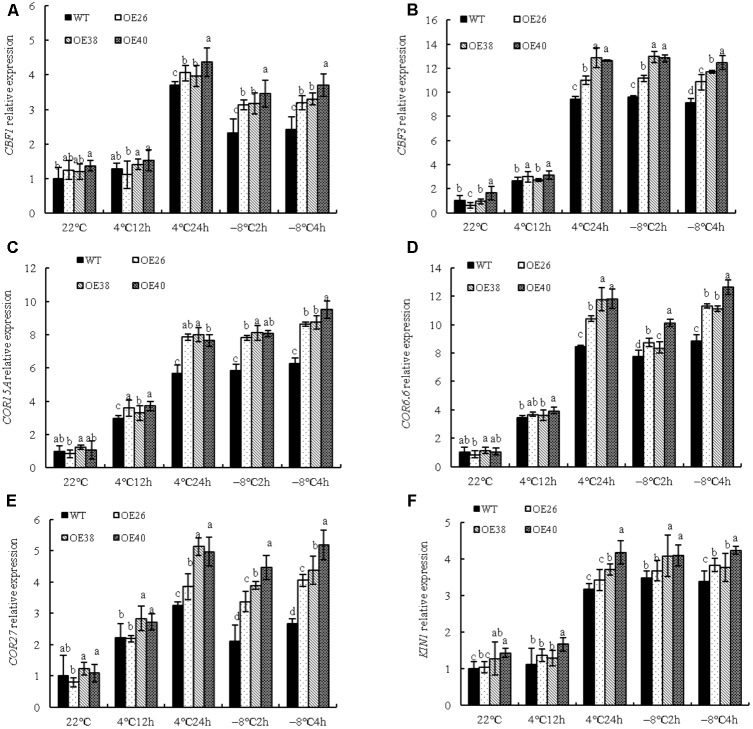
Effects of low temperature on cold related genes expression of *VvBAP1* ectopic overexpressing plant leaves. Relative expression of cold related genes *CBF1* **(A)**, *CBF3* **(B)**, *COR15A* **(C)**, *COR6.6* **(D)**, *COR27* **(E)**, and *KIN1* **(F)** in the leaves of overexpressing lines with the 4 and -8°C treatment. Three independent experimental replications were conducted. Values are the means ± SE of three independent experiments (*P* < 0.05). Lower-case letters above bars denote significant differences attested by Tukey′s HSD test.

The pathway of plant responses to low temperature can be categorized into *C-repeat Binding Factor* (CBF) pathways and non-CBF pathways. To explore whether *VvBAP1* is involved in the cold resistant process through the CBF pathway in grapes, the expression of cold-responsive signal genes in *VvBAP1* over-expressing *Arabidopsis* was tested under low temperature. Cold-related gene expression in *VvBAP1* over-expressing plants, including that of *CBF1*, *CBF3*, *COR15A*, *COR6.6*, *COR27*, and *KIN1* were not changed remarkably under normal temperature, but were higher than those of WT plants after low-temperature treatment (**Figures 6A–F**). They were all highly activated at 4°C after 24 h of treatment and specifically induced by -8°C treatment. Therefore, we speculated that *VvBAP1* may improve cold resistance through increasing the expression of CBF and downstream genes in plants.

## Discussion

The majority of commercial grape cultivars belong to the European grape. While these cultivars have excellent organoleptic qualities, they suffer relatively poor tolerance to the cold experienced during winter and cold snaps during spring, resulting in significant damage to grapevines growing (Yu et al., 2017). Thus studying the cold resistance mechanism in grapes is an important research task that provides a pre-stage foundation for grape breeding. BAP1 is a phospholipid-binding protein with a C2 structural domain; its increased expression can be induced in *Arabidopsis* by low temperature (Yang et al., 2006; Zhu et al., 2011). In our previous work, the full-length cDNA of *VvBAP1* was cloned from leaves of *V. vinifera* cultivar ‘F-242’ tissue culture seedlings. Bioinformatic analysis indicated that VvBAP1 consists of 531 nucleotides, encoding 176 amino acids with the conserved C2 domain (Zhang et al., 2014). To study the possible involvement of this gene in the cold resistant process, the cold-resistant varieties ‘F-242’ and ‘Zuoyouhong’ and the cold-sensitive varieties ‘Cabernet Sauvignon’ and ‘Chardonnay’ were selected to test the expression difference of *VvBAP1*. The transcriptional patterns of *VvBAP1* in cold-resistant grapes was significantly higher than that in cold-sensitive grapes (**Figure 1A**). Furthermore, qRT-PCR and the GUS staining technique were applied to analyze *VvBAP1* expression, and the results indicated that low temperature and the signal molecule Ca^2+^ could significantly induce the transcription of this gene (**Figures 1B,C**), indicating that *VvBAP1* may play a role in activating the cold stress response in grape.

To confirm the biological function of VvBAP1, the *VvBAP1* gene was transferred to *Arabidopsis* and three homozygous lines were obtained. After low-temperature acclimation at 4°C and low temperature treatment at -8°C, the recovery capability of three transgenic lines (OE26, OE38, and OE40) was significantly superior to that of the WT, and they showed a significantly higher survival rate (**Figures 2A–C**). The transcription factor AtICE1 involved in the cold resistance in *Arabidopsis* can be bound with the MYC cis-acting element upstream of *AtBAP1* to induce *AtBAP1* expression, as a response to cold stress (Zhu et al., 2011). This experiment has shown that the *VvBAP1* gene is involved in the process of cold resistance in grapes.

This experiment further studied the molecular mechanism with which VvBAP1 responds to low temperature. Under cold stress, ROS content in plants is increased, which damages lipid oxidation, protein, and DNA, and severely impacts both growth and development. Plants can remove excessive ROS via antioxidant enzymes (including SOD and POD). For instance, chrysanthemum can strengthen its cold resistance through enhancing the activity and gene expression of antioxidant enzymes, including SOD, CAT, and APX (Chen et al., 2014). Low temperature is known to induce the activity and gene expression of Cu/Zn-SOD in cassava, and the expression of native cytosolic SOD, while simultaneously activating the antioxidative defense mechanisms via cyclic ROS scavenging, thus improving its tolerance to cold stress (Xu et al., 2014a). This experiment utilized *VvBAP1* overexpressing *Arabidopsis* and found that the activities of SOD and POD in transgenic plants were significantly higher than those of WT plants, as were the expressions of *Cu/ZnSOD* and *PRX34* (**Figures 3A–D**). *AtBAP1* and *AtZAT10* in *Arabidopsis* can function as signal genes in the process of ROS signal transduction from the chloroplast to the nucleus, in response to cold stress (van Buer et al., 2016). The results of the current experiment suggest that VvBAP1 in grapes can also be involved in the cold resistance process, through activating antioxidant enzyme activities. Whether BAP1 adjusts enzyme activity directly or indirectly, the relationship between BAP1 and transcription factor ZAT10 that are involved in the process of oxidative stress, and whether they complete the adjustment of the antioxidant enzyme together, will be investigated in follow-up experiments. The damage to the cytomembrane caused by low temperatures can be expressed via cell membrane permeability and MDA content. The results of this experiment showed significantly lower cell membrane permeability and MDA content of transgenic plants compared to WT plants (**Figures 3E,F**), demonstrating that VvBAP1 can improve the stability of the cytomembrane to further improve the cold resistance of ectopic overexpressing plants.

*Vitis vinifera* cultivars undergo a generally conserved and highly coordinated metabolic shift during their stress responses (Hochberg et al., 2013). These metabolites include amino acids, soluble sugar, tartaric acid, etc. Soluble sugars, including sucrose, fructose, glucose, maltose, and raffinose, are important osmotic adjustment substances, and play a crucial role in the plant response to stress (Tarkowski and Van den Ende, 2015). When plants are under cold stress, the soluble sugar is significantly increased. Sugars play multiple roles in low temperature tolerance. As typical compatible osmolytes, they contribute to the preservation of water within plant cells, thereby reducing water availability for ice nucleation in the apoplast (Markovskaya et al., 2010). In recent research, the sucrose phosphate synthase (SPS) activity decreased in the SPSA1 mutant of *Arabidopsis*, which resulted in an increase of starch synthesis (Sun et al., 2011); the activities of acid invertase and SS in *Cerasus pseudocerasus* under cold stress were significantly increased (Turhan and Ergin, 2012). Low temperature can induce the expression of *TaSS* and *TaSPS* and increase the soluble sugar content (Zeng et al., 2011). Furthermore, starch grain in plant cells can be decomposed into sucrose, glucose, maltose, and other products, under the catalysis of α-amylase (AMY) and β-amylase (BMY or BAM). Maltose content and *BMY3* relative expression are increased to some extent in *Arabidopsis* leaves after 24 h of low-temperature treatment (Sicher, 2011). RNAi interference can be utilized to reduce the expression of *AtBMY8* genes in *Arabidopsis*, restrain the accumulation of maltose in cold shock, and strengthen the sensitivity of PSII photochemistry activities with cold damage (Kaplan and Guy, 2005). An interesting phenomenon was found during the growth process of *VvBAP1* ectopic overexpressing *Arabidopsis* plants: the stems and leaves of transgenic plants more rapidly incurred aphids compared to WT plants, which was likely due to the high soluble sugar content of transgenic lines. Therefore, we tested the soluble sugar content in the leaves of *VvBAP1* ectopic overexpressing plants and found that the soluble sugar content was higher than in WT plants to some extent under normal conditions, and significantly higher after low-temperature treatment (**Figure 4A**). The activity of enzymes related to the soluble sugar metabolism under low temperature (BAM, SS, and G6PDH) was significantly higher than in WT plants (**Figures 4B–D**). Furthermore, the expression of *BAM4*, *BAM5*, *BAM6*, *BAM7*, *SS4*, *and G6PD5* was significantly increased in *VvBAP1* ectopic overexpressing plants compared to WT plants (**Figures 5A–F**). The relationship between BAP1 and soluble sugars has not yet been reported. Our results showed that *VvBAP1* can increase the soluble sugar content of transgenic plants under low temperature. However, how VvBAP1 improves the sugar content remains unknown and will be the focus of future research. It has been reported that the CBF transcription factor could regulate the *PtrBAM1* gene expression in *Poncirus trifoliata*, thus increasing the soluble sugar content and enhancing the cold resistance (Peng et al., 2014). Thus, the possible coordination between VvBAP1 and other transcription factors to adjust the soluble sugar content is worthy of further research.

Cold stress related genes, including *CBF3*, *COR15A*, and *COR6.6* participate in the response to low temperature. The experimental results mentioned above indicated the involvement of VvBAP1 in cold stress. However, the relationship between these genes remains unknown. In *VvBAP1* ectopic overexpressing *Arabidopsis*, the relative expression of the cold-related genes *CBF1*, *CBF3*, *COR15A*, *COR6.6*, *COR27*, and *KIN1* were higher than those of WT plants (**Figures 6A–F**). Therefore, *VvBAP1* in grapes may be involved in the response to low temperature. Unlike *AtBAP1* in *Arabidopsis*, the *VvBAP1* promoter does not have a cis-acting element for binding with transcription factor ICE1, but instead has the element (MBS) for binding with the transcription factor MYB (Supplementary Figure 4). Further research is required to determine which transcription factors are adjusting VvBAP1, whether any interaction takes place with the cold signal pathway ICE1-CBF-COR, as well as the detailed mechanism for the involvement of VvBAP1 in the process of cold stress.

In summary, our findings confirm that *VvBAP1* could enhance the cold resistance in the grapevine through regulating the sugar content and activating antioxidant enzyme activity. It is of significance to further study the function and mechanisms of *VvBAP1* genes in the grapevine.

## Author Contributions

LH designed and performed the experiments, interpreted the data, and wrote the article. XL designed the experiments and edited the article. GZ and FZ performed the experiments and interpreted the data. DZ and ZZ analyzed the data. All authors read and approved the manuscript.

## Conflict of Interest Statement

The authors declare that the research was conducted in the absence of any commercial or financial relationships that could be construed as a potential conflict of interest.
