# Supplementary material for: VvBAP1 Is Involved in Cold Tolerance in Vitis vinifera L
Source: Front Plant Sci. 2018 Jun 18;9:726. doi: 10.3389/fpls.2018.00726 (PMC6016009; doi:10.3389/fpls.2018.00726)
Supplement: Supplementary file 1 [file Image_1.pdf]

Supplementary Material

Table 1 Real-time PCR primers

Gene name	Gene accession ID	Primer sequence (5' to 3')
VvBAP1	VIT_15s0048g020709 (GGBD 12X V1) (100248930)	FP: AGAAGAATGCCTTTGCCTTG<br>RP: CCTCGCAGTTCCGATGACCC
VvACTIN	VIT_00026580001 (GGBD 12X V1) (100232866)	FP: AATGAGAGATGGCTGGAAGAG<br>RP: TACGAGCAAGAGCTGGAAA
AtBAM4	AT5G55700 (835664)	FP: GCTTCCGTCTTGTCTCGTCA<br>RP:TGCCTTAGTCCCATCTCATC
AtBAM5	AT4G15210 (827185)	FP: TGAGCAAGGCATGGAAAG<br>RP: CGGATAACCGAAGGTAAGT
AtBAM6	AT2G32290 (817789)	FP:GATCATACTAAACGCAAGGCCT<br>RP: GCAGCTCGTGATTATACCTT
AtBAM7	AT2G45880 (819196)	FP:GGGGTTAAAGTGGATTGTT<br>RP:TGGTTCGTCCTATTTCTGCC
AtSS4	AT4G18240 (827550)	FP: ACAGAGTGAGAAACGGTGAG<br>RP: GCCATTACAAGTAGCGAAA
AtG6PD5	AT3G27300 (822349）	*FP: ATGAAAGATGGGAAGGTGTT*<br>*RP: CAAGAATAAGGCGTTCGTAA*
AtCBF1	AT4G25490 (828653)	*FP: CGGCTACTACTAACCAGACCC*<br>*RP: AGCCTTAGCACCACGAAAAC*
AtCBF3	AT4G25480 (828652)	*FP: TAAGTGGGTTTGTGAGGTTAGAG*<br>*RP: CACACATCTCATCCTGAAACG*
AtCOR15A	AT2G42540 (818854)	*FP: AACTCAGTTCGTCGTCGTTT*<br>*RP: CATCTGCTAATGCCTCTTTTG*
AtCOR6.6	AT5G15970 (831454)	*FP: TTGAAGACGGAACAGAGC*<br>*RP: CAAACAACGGTCAACTGG*
AtCOR27	AT5G42900 (834301)	*FP:TTACAACATCAACGCCCACT*<br>*RP:TCACCACCGACGAAGAATC*
AtKIN1	AT5G15960 (831453)	*FP: CATCAACGCCCACTGCTT*<br>*RP:ACGACATCCATCACCACCG*
AtACTIN	AT3G18780 (821411)	*FP: GGTAACATTGTGCTCAGTGGTGG*<br>*RP: CACGACCTTAATCTTCATGCTGC*

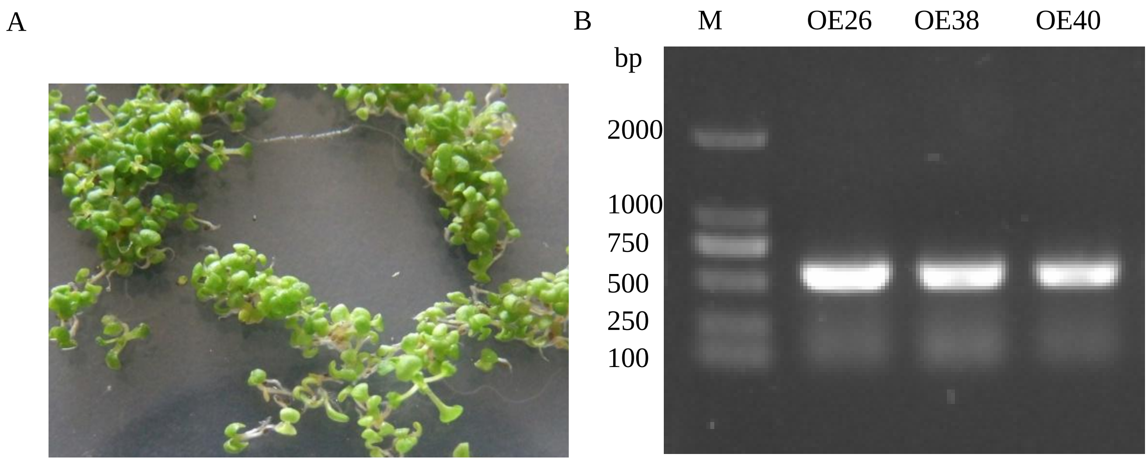

Supplementary Figure 1. The growth of *VvBAP1* over-expression *Arabidopsis* on MS-Hyg medium (A) and identification of *VvBAP1* over-expressing *Arabidopsis* by PCR (B).

A

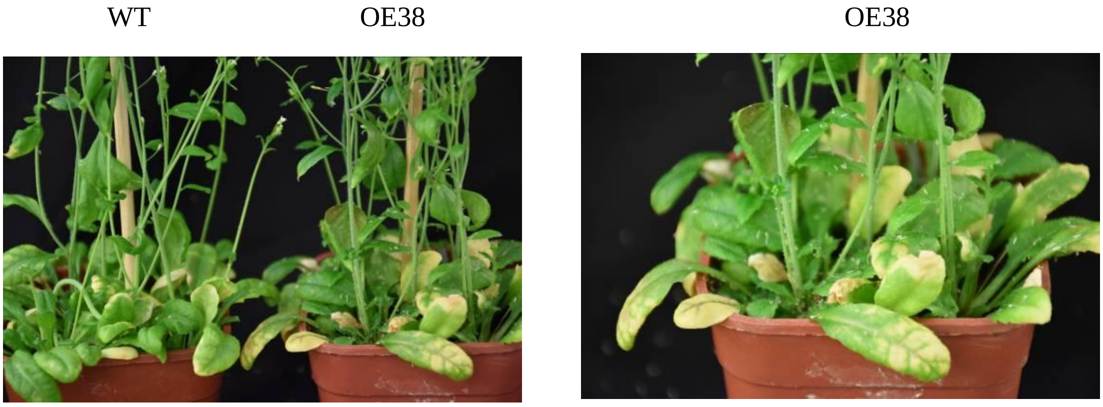

B

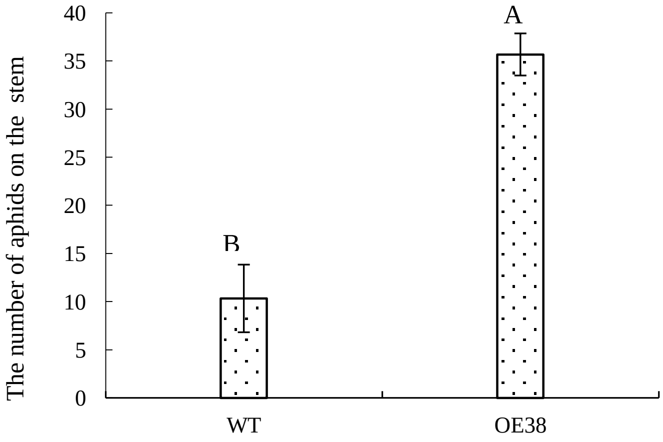

Supplementary Figure 2. The phenotype of *VvBAP1* overexpressing *Arabidopsis* (A) and the number of aphids on the stems of *VvBAP1* overexpressing *Arabidopsis* (B). Values are the means ± SE of three independent experiments. Upper case letters above bars denote significant differences ($P < 0.01$) attested by Tukey's HSD test.

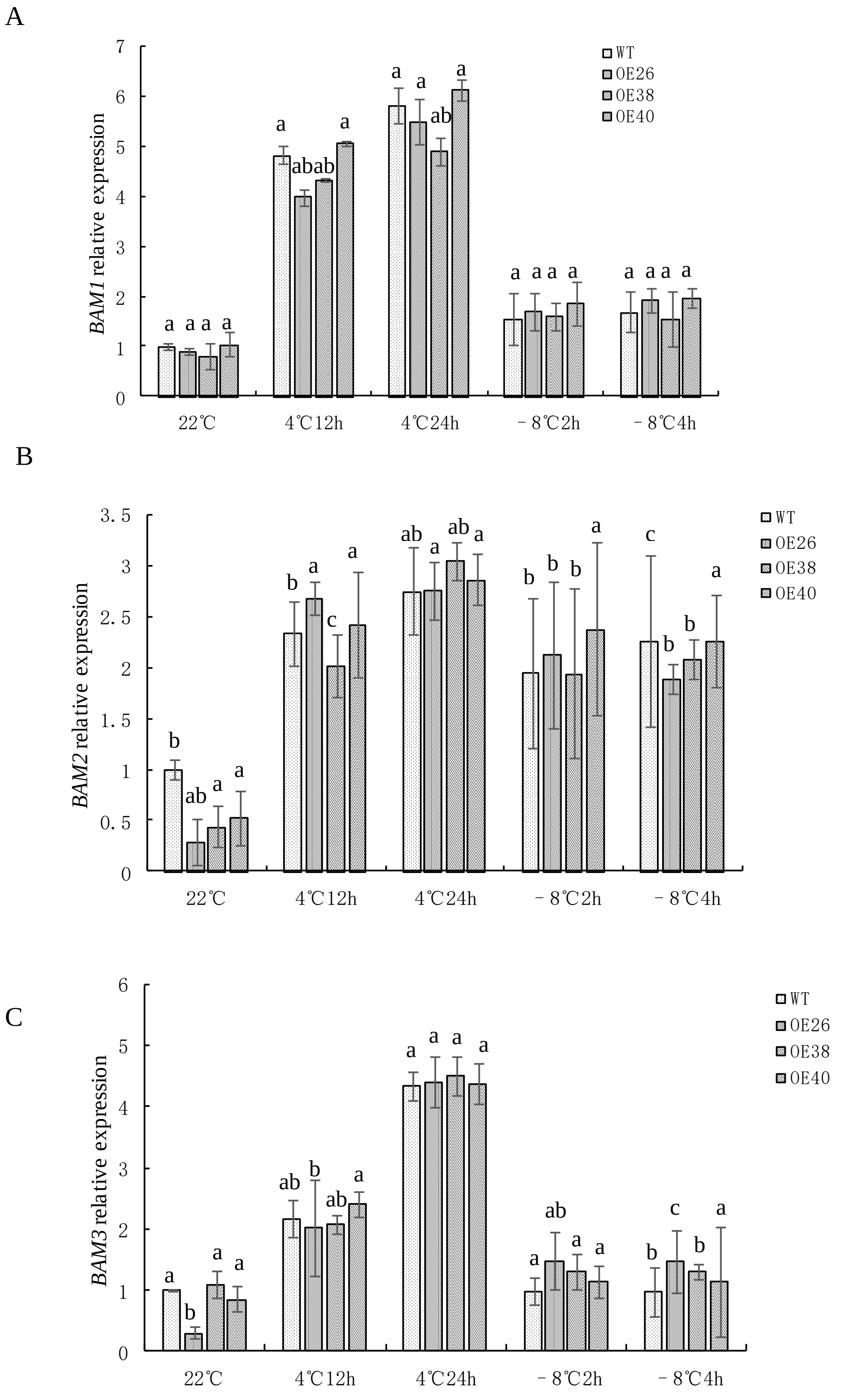
A
BAM1 relative expression
WT
OE26
OE38
OE40
22℃
4℃12h
4℃24h
－8℃2h
－8℃4h
B
BAM2 relative expression
WT
OE26
OE38
OE40
22℃
4℃12h
4℃24h
－8℃2h
－8℃4h
C
BAM3 relative expression
WT
OE26
OE38
OE40
22℃
4℃12h
4℃24h
－8℃2h
－8℃4h

E

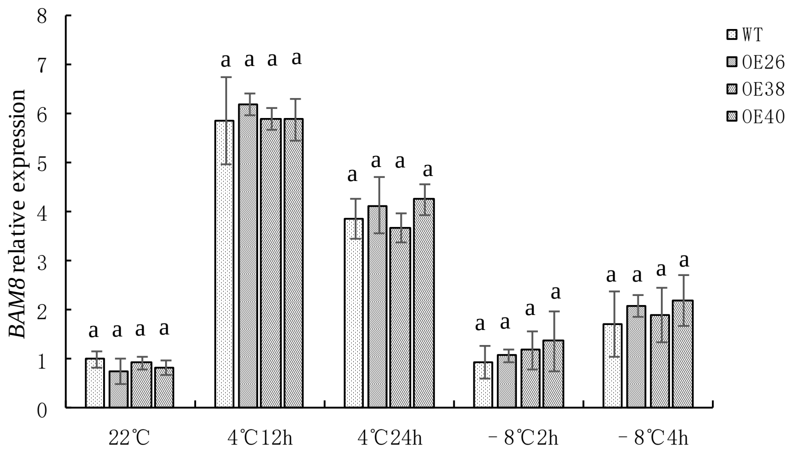

F

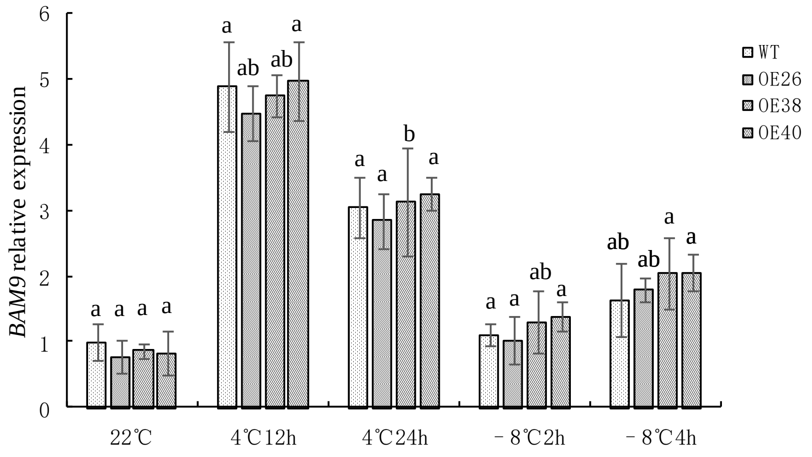

Supplementary Figure 3. Effects of low temperature on *BAM1* (A) , *BAM2* (B) , *BAM3* (C) , *BAM8* (D) , *BAM9* (E) gene expression of *VvBAP1* ectopic over-expression plant leaves. Three independent experimental replications were conducted. Values are the means ± SE of three independent experiments (P < 0.05). Lower-case letters above bars denote significant differences attested by Tukey's HSD test.

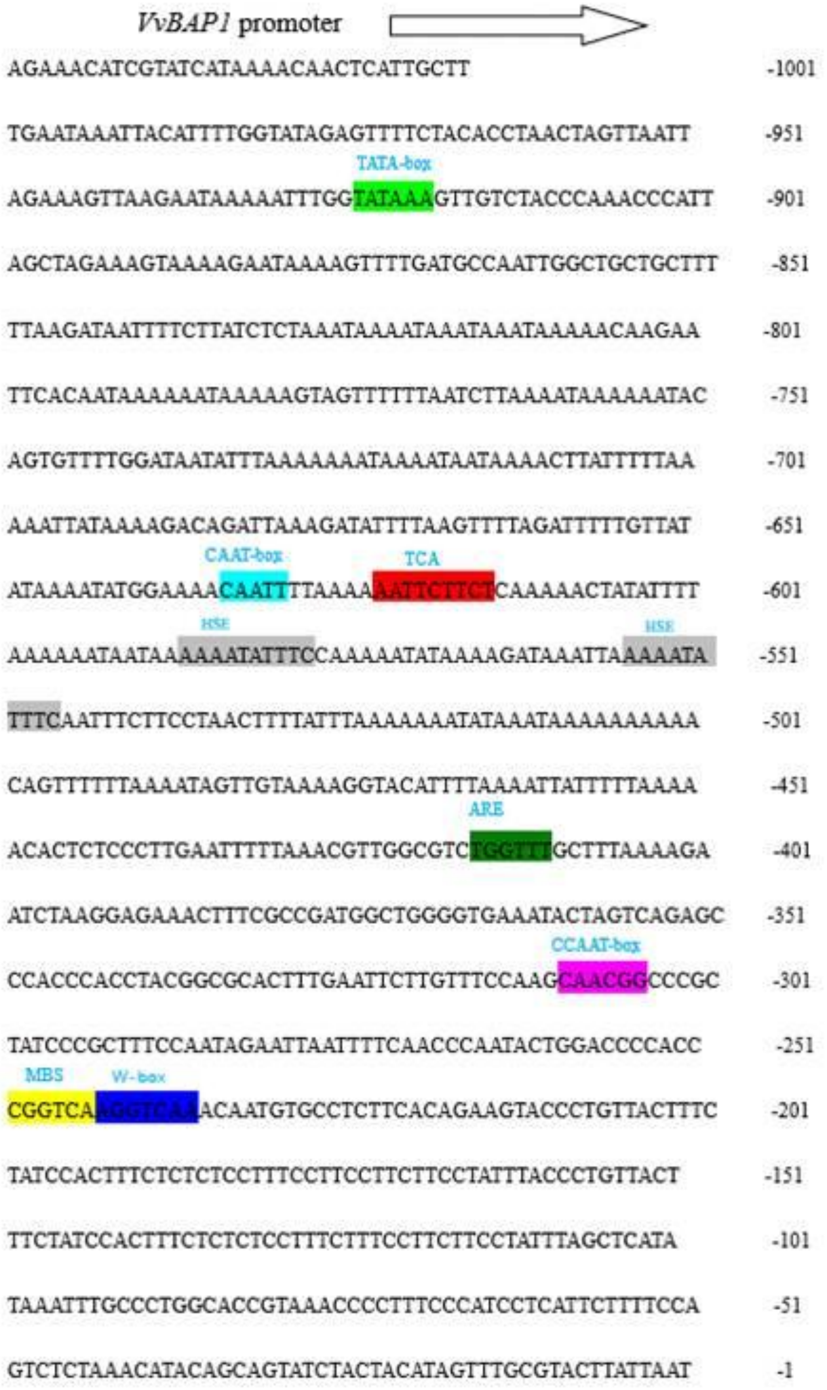

Supplementary Figure 4. The sequence and analysis of functional elements of *VvBAP1* promoter
